# Anti-factor H antibody-positive C3 glomerulonephritis secondary to poststreptococcal acute glomerulonephritis with diabetic nephropathy

**DOI:** 10.1007/s13730-023-00809-3

**Published:** 2023-07-15

**Authors:** Yuki Oba, Hiroki Mizuno, Sekiko Taneda, Toshihiro Sawai, Takashi Oda, Daisuke Ikuma, Masayuki Yamanouchi, Tatsuya Suwabe, Kei Kono, Keiichi Kinowaki, Kenichi Ohashi, Naoki Sawa, Yoshifumi Ubara

**Affiliations:** 1 Nephrology Center, Toranomon Hospital Kajigaya, 1-3-1 Kajigaya, Kawasaki, Kanagawa 213-8587 Japan; 2 Department of Pathology, Tokyo Women’s Medical University, 8-1 Kawada-Cho, Shinjuku-Ku, Tokyo, 162-8666 Japan; 3 Department of Pediatrics, Shiga University of Medical Science, Seta Tsukinowa-Cho, Otsu, Shiga 520-2192 Japan; 4 Department of Nephrology and Blood Purification, Tokyo Medical University Hachioji Medical Center, 1163 Tatemachi, Hachioji, Tokyo 193-0998 Japan; 5 Department of Pathology, Toranomon Hospital, 2-2-2 Toranomon, Minato-Ku, Tokyo, 105-8470 Japan; 6 Department of Human Pathology, Tokyo Medical Dental University, 1-5-45 Yushima, Bunkyo-Ku, Tokyo, 113-8510 Japan

**Keywords:** Diabetes mellitus, Infection-related glomerulonephritis, Poststreptococcal glomerulonephritis, C3 glomerulonephritis, Anti-factor H antibody

## Abstract

Poststreptococcal acute kidney glomerulonephritis (PSAGN) has been seen in adults in recent years, especially in patients with type 2 diabetes mellitus, and the renal prognosis has not always been good. There have been cases of PSAGN in which complete remission was not achieved and hematuria and proteinuria persisted, leading to end-stage renal disease. Previous reports showed that the patients subjected to PSAGN have an underlying defect in regulating the alternative pathway of complement, and they identified that antibodies to the C3 convertase, C3 nephritic factors (C3NeF), are involved. C3NeF stabilizes C3 convertase, sustains C3 activation, and causes C3 glomerulonephritis (C3GN). On the other hand, factor H is a glycoprotein that suppresses the overactivation of the alternative pathway by decaying the C3 convertase. Anti-factor H (aFH) antibodies interfere with factor H and cause the same activation of the alternative pathway as C3NeF. However, a limited number of reports describe the clinical course of C3GN with aFH antibodies. We encountered a 49-year-old Japanese man with type 2 diabetes mellitus. He was referred to our hospital because of his elevated serum creatinine, proteinuria, hematuria, and developed edema on both legs. He was diagnosed as PSAGN at the first kidney biopsy, and his renal function improved and edema and hematuria disappeared, but proteinuria persisted after 5 months. He was diagnosed as C3GN at the second kidney biopsy. In our case, no C3NeF was detected. However, a high titer of aFH antibodies was detected in stored serum from the initial presentation, providing a unified diagnosis of aFH antibody-positive C3GN secondary to PSAGN. He progressed to end-stage renal disease (ESRD) and hemodialysis was started. The persistence of high levels of aFH autoantibodies may have caused C3GN secondary to PSAGN due to activating the alternative complement pathway, which eventually worsened the nephropathy and led to ESRD.

## Introduction

Poststreptococcal acute kidney glomerulonephritis (PSAGN) is an acute endocapillary proliferative glomerulonephritis triggered by a streptococcus species. It is common in childhood and is considered to have quite a good renal prognosis. However, in recent years this disease has also been seen in adults due to the frequency and severity of infections in the elderly and the higher prevalence of diabetes, the existence of which is an underlying condition predisposing to infection [1, 1], and the renal prognosis has not always been good [2]. In addition, PSAGN has been found to have a genetic background, and reports have described cases of conversion to C3 glomerulonephritis (C3GN) via the activated alternative complement pathway [3].

Here, we show a rare case of anti-factor H autoantibody-positive C3GN, a rare type of GN, which was developed in a patient with PSAGN and diabetic nephropathy, and we also present its histopathological transformation with time by repeat biopsy.

## Case presentation

A 49-year-old Japanese man was admitted to our hospital for evaluation of a decline in renal function and edema. Type 2 diabetes was noted at age 42 years. At age 46, the patient underwent surgical treatment for diabetic foot gangrene and laser treatment for diabetic retinopathy. The patient’s body weight was reduced from 115 to 90 kg by strict diet control, and hemoglobin A1c normalized from around 10% to 5.4% without hypoglycemic agents. At that time, the serum creatinine level was 0.9 to 1.2 mg/dL and urine protein excretion was 1.77 g/day. The patient had not seen a medical service since then. One month before admission to our hospital, the patient had a fever and went to see his previous doctor. Urinalysis showed pyuria and the patient was treated with ceftriaxone as a urinary tract infection, and the fever resolved. However, signs of acute kidney injury emerged: Serum creatinine increased to 4.3 mg/dL within a few days and edema developed on both legs. Therefore, the patient was referred to our hospital.

At admission, the patient was 169 cm tall and weighed 85.8 kg. His blood pressure was 178/84 mm Hg, and his temperature, was 36.7 °C. Marked pitting edema was observed on both legs. Neurological examination showed a decreased vibration sensation (6 s/6 s), and numbness was noted in the soles of both feet.

The laboratory data showed hypoalbuminemia (albumin, 1.9 g/dL) and acute kidney injury (urea nitrogen, 42 mg/dL; creatinine, 3.35 mg/dL) (Table 1). Serum C-reactive protein (CRP) was high (6.3 mg/dL). Serum complement titers were low (CH50, 37 U/mL; C3, 35.0 mg/dL; and C4, 34.0 mg/dL). The anti-streptolysin O (ASO) concentration was 1424 IU/mL. Blood culture tests could not identify any significant causative organisms. Twenty-four-hour urinary protein excretion was 7.33 g, and the urine sediment contained 11 to 30 red cells and less than 1 white cell per high-power field.

**Table 1:** Laboratory results at first and second admission

Laboratory test	Reference range	Results at the first kidney biopsy	Results at the second kidney biopsy
Blood analysis			
UN, mg/dL	8.0–20.0	42	29
Creatinine, mg/dL	0.46–0.79	3.35	1.75
eGFR, mL/min	> 89	16.9	34.4
Sodium, mmol/L	138–145	145	145
Potassium, mmol/L	3.6–4.8	4.2	3.8
Chloride, mmol/L	101–108	109	108
Calcium, mg/dL	8.8–10.1	7.5	8.1
Phosphate, mg/dL	2.7–4.6	4.1	3.4
Uric acid, mg/dL	2.6–7.0	11.6	10.5
IgG, mg/dL	861–1747	2264	974
IgA, mg/dL	93–393	573	429
IgM, mg/dL	50–269	74.1	63.1
CH50, IU/mL	31–58	37	60
C3, mg/dL	73–138	35	122
C4, mg/dL	11–31	34	32
Albumin, g/dL	4.1–5.1	1.9	2.7
AST, IU/L	13–30	14	41
Bilirubin, total, mg/dL	< 1.5	0.2	0.7
CRP, mg/dL	< 0.14	6.3	0.1
Ferritin, ng/mL	5–120	393	296
ASO, IU/mL	< 239	1424	237
WBC count	4–11 × 10^3^/μL	7.5	9.4
Hemoglobin, g/dL	12.0–16.0	7.2	11.1
Platelet count	120–450 × 10^3^/μL	194	275
Hemoglobin A1c, %	4.0–5.6	5.1	4.6
Urinary analysis			
pH	4.5–8.0	6.0	6.0
Specific gravity	1.002–1.030	1.023	1.011
RBC	< 4/HPF	11–30	< 4
WBC	< 4/HPF	> 100	< 1
Urine protein, g/gCre	< 0.3	12.8	7.33
Amorphous crystals	None seen, rare, occational /HPF	None seen	None seen
Bacteria	None seen, rare, occational /HPF	None seen	None seen
Nitrate	Negative	Negative	Negative

*UN* urea nitrogen, *eGFR* estimated glomerular filtration rate, *Ig* immunoglobulin, *AST* aspartate aminotransferase, *CRP* C-reactive protein, *ASO* anti-streptolysin O, *WBC* white blood cells, *RBC* red blood cells

To evaluate the nephrotic syndrome and acute kidney injury, an echo-guided percutaneous kidney biopsy of the right kidney was performed.

## Results of the first kidney biopsy

**Fig. 1:**
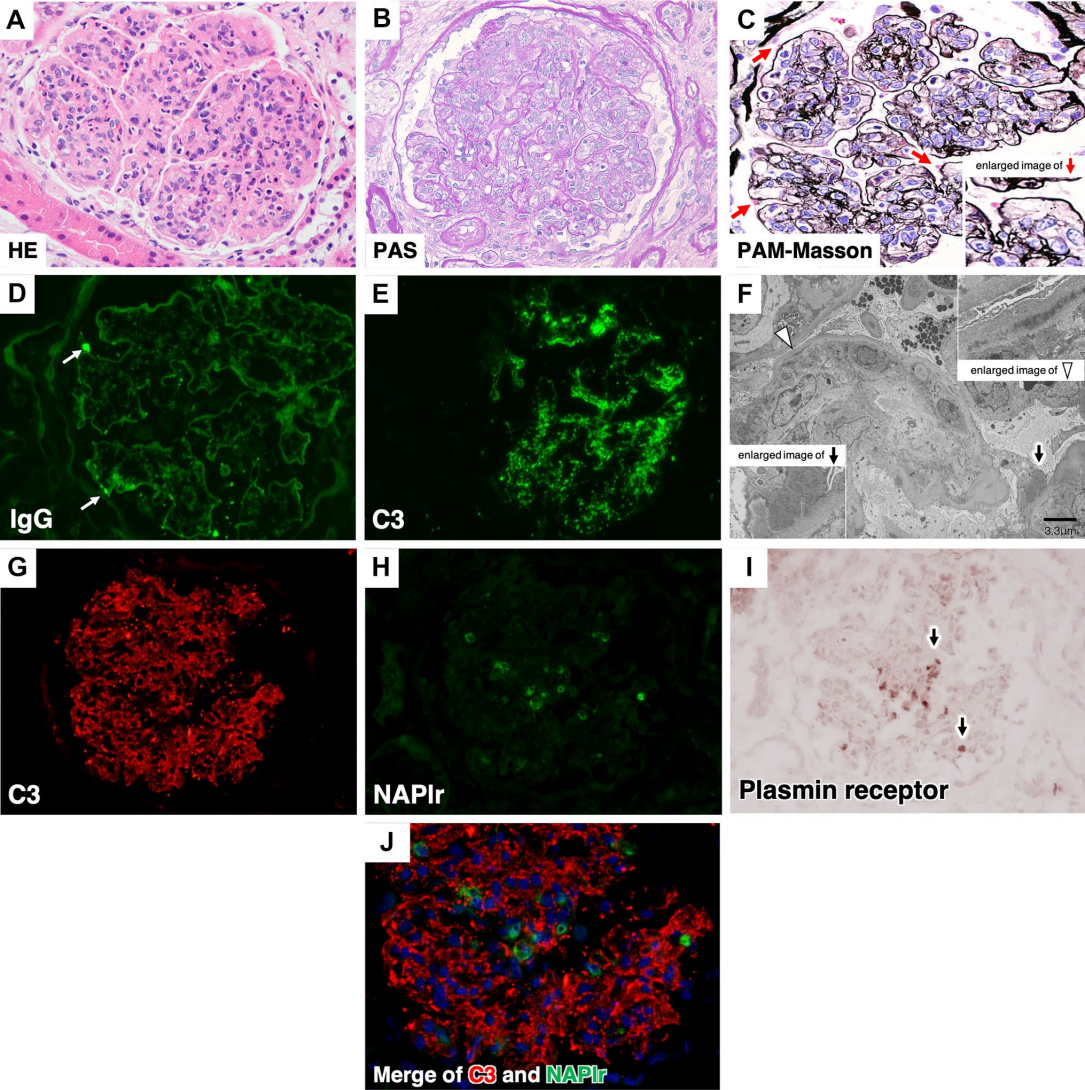
Results of the first kidney biopsy. **A** Light microscopy of hematoxylin–eosin staining. It showed diffuse hypercellularity with a lobulated structure, invasion of numerous neutrophils into the glomerular capillary tuft, increased mesangial matrix, and mesangial hypercellularity. **B** Periodic acid Schiff (PAS) staining. As light microscopy shows, diffuse hypercellularity in the glomerular capillary tuft. **C** Periodic acid-methenamine-silver stain-Masson staining. Swollen endothelial cells and subepithelial humps (large deposits on the glomerular basement membrane [GBM], red arrowhead) were noted. **D** Immunofluorescence staining of IgG. Scattered granular staining (arrow) and mild linear staining for IgG along the GBM were shown. **E** Immunofluorescence staining of C3. Granular staining of C3 was observed along the GBM and in the mesangial region (referred to as the starry sky or garland pattern). **F:** Electron microscopy. It showed fine granular electron-dense deposits (arrowhead) arranged in a row inside the basement membrane and subepithelial humps (arrow), as well as endocapillary hypercellularity with the invasion of neutrophils and macrophages into the glomerular capillary tuft. **G** Immunohistological staining of C3 when stained for NAPlr and plasmin receptor. C3 was positive, as Fig. 1E shows. **H** Immunofluorescence staining of nephritis-associated plasmin receptor (NAPlr). NAPlr was positive within the GBM. (green). **I** Immunohistochemistry staining of plasmin receptor. Activated plasmin was stained in mesangial cells and glomerular neutrophils and endothelial cells. (arrow). **J** Immunohistochemistry staining of C3 and NAPlr. Merging of the C3 (red) and NAPlr (green) images showed that NAPlr was present in an area within the GBM that was different from the area with C3 deposition

Light microscopy (LM) showed global sclerosis in 5 of 25 glomeruli. The preserved glomeruli were diffusely enlarged and showed diffuse endocapillary hypercellularity with a lobulated structure, invasion of numerous neutrophils, increased mesangial cells and matrix, and swollen endothelial cells, as well as subepithelial humps (large deposits on the GBM; Fig. 1A–C). Interstitial fibrosis and tubular atrophy occupied less than 15% of the total kidney cortical area. However, arteriolar hyalinosis was mild, and some polar vasculosis was found at the vascular pole of glomeruli.

Immunofluorescence (IF) staining showed scattered granular and mild linear deposits that stained 1 + for IgG along the glomerular basement membrane (GBM) (Fig. 1D) and granular deposits that stained 2 + for C3 along the GBM and mesangial region (Fig. 1E and G).

Electron microscopy (EM) showed fine granular electron-dense deposits (EDDs) arranged in a row inside the GBM and subepithelial humps on the GBM, as well as endocapillary hypercellularity with the invasion of neutrophils and macrophages into the glomerular capillary tuft (Fig. 1F). The GBM was thickened to a width of 650 to 970 nm.

Immunofluorescence staining of nephritis-associated plasmin receptor (NAPlr) (Fig. 1H) and immunohistological staining of plasmin receptors were stained (Fig. 1I) in an area within the GBM that was different from the area with deposition of C3 (Fig. 1J) [4, 5].

## Diagnosis and Clinical Follow-up after the first biopsy

PSAGN was the most reliable diagnosis, but diabetic nephropathy class 1 or 2a was also diagnosed according to Tervaert’s pathologic classification of diabetic nephropathy. The positive staining of both NAPlr and plasmin receptors provided additional support for diagnosing PSAGN with diabetic nephropathy [4, 5].

After the first kidney biopsy, the patient’s water retention and hypertension were strictly controlled by diuretics and antihypertensive agents, a strict salt diet (less than 6 g daily), and water restriction (less than 1000 mL daily). Body weight consequently decreased from 85.6 kg to 71.7 kg and proteinuria, from 12.8 g/day to 2.97 g/day. Within a month, serum creatinine also improved from 3.35 mg/dL to 2.01 mg/dL. It was thought that continuing body weight control and antihypertensive treatment would improve his creatinine and proteinuria caused by PSAGN with diabetic nephropathy, so no immunosuppressive therapy was given. However, he interrupted his hospital visit and came back three months after his discharge. The patient’s body weight and proteinuria had increased to 83.9 kg and 13.99 g/day, respectively; the laboratory data are shown on the right column of Table 1. Serum creatinine was 1.75 mg/dL; CRP, 0.1 mg/dL; serum C3, 122.0 mg/dL; serum C4, 32.0 mg/dL; CH50, 60 U/mL; and ASO, 237 IU/mL. A second kidney biopsy was performed five months after the first biopsy to elucidate why the nephrotic syndrome had worsened again.

## Results of the second kidney biopsy

**Fig. 2:**
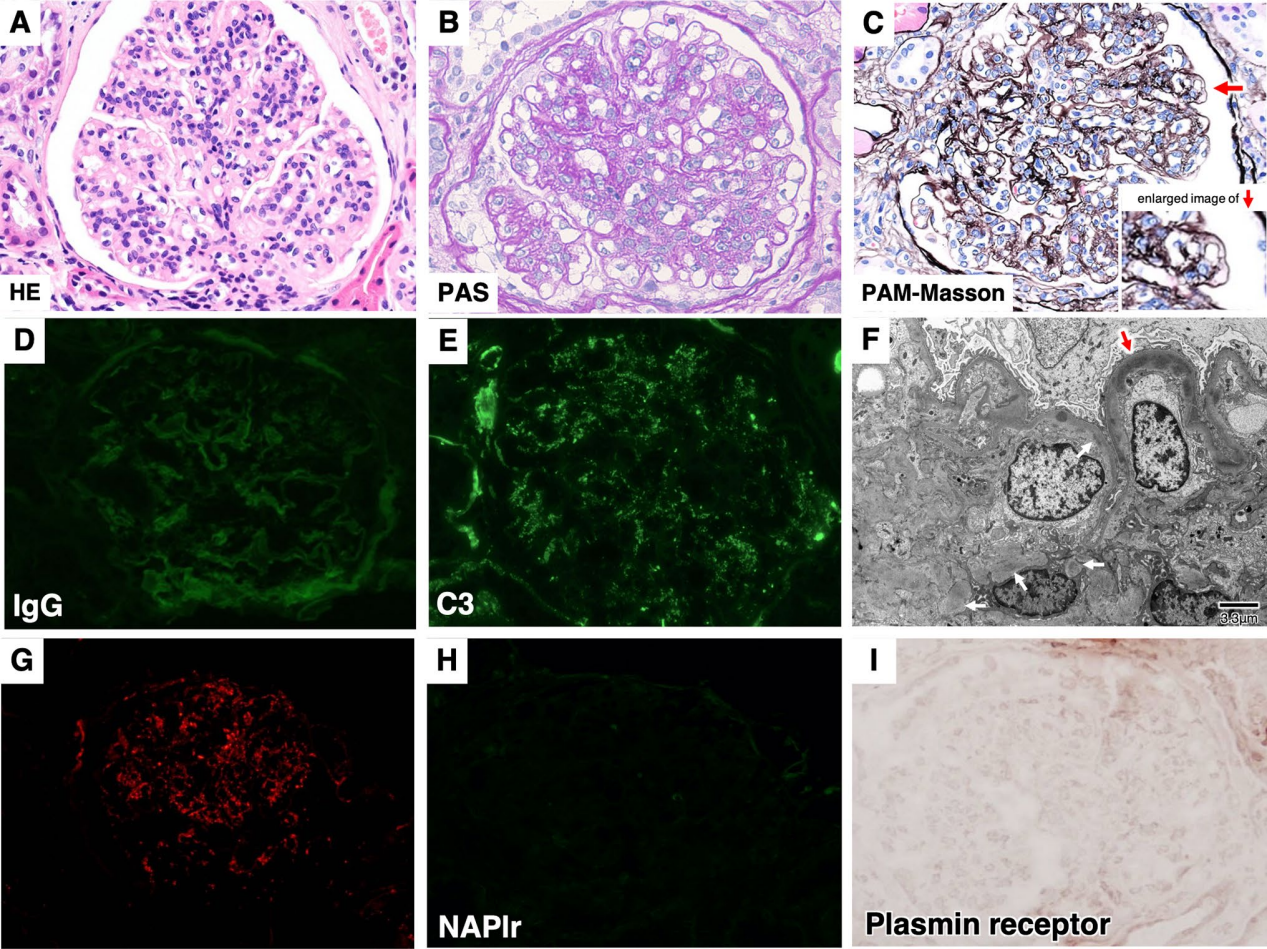
Results of the second kidney biopsy.** A** Light microscopy of hematoxylin–eosin staining. Diffuse hypercellularity of the preserved glomeruli was still present but milder, and invasion of neutrophils into the glomerular capillary tuft was less noticeable. **B** Periodic acid Schiff (PAS) staining. As light microscopy shows, diffuse hypercellularity in the glomerular capillary tuft but less than Fig. 1B, that of the first kidney biopsy. **C** Periodic acid-methenamine-silver stain-Masson (PAM-Masson) staining showing duplication of the glomerular basement membrane. **D** Immunofluorescence staining of IgG. IgG staining was less severe than that of the first kidney biopsy. **E** Immunofluorescence staining of C3. Not IgG, but only C3 granular staining was still present. **F** Electron microscopy. Subendothelial electron-dense deposits (black arrow) were more prominent and could also be seen in mesangial areas (white arrow). **G–I** Immunohistological staining of C3 (G), nephritis-associated plasmin receptor(NAPlr) (H), and plasmin receptor (I) at the same time. As Fig. 2E shows, C3 was still present, but unlike Fig. 1H and, NAPlr and Plasmin activity became both negative

On LM, global sclerosis was present in 19 of 38 glomeruli. Diffuse hypercellularity of the preserved glomeruli was still present, but it was milder, and the invasion of neutrophils into the glomerular capillary tuft was less noticeable than that of first kidney biopsy (Fig. 2A, B). Duplication of the GBM was apparent (Fig. 2C). IF showed less IgG deposits that stained ± (Fig. 2D); however, C3 granular deposits (Fig. 2E, G) were still present that stained 1 + on the GBM. EM showed that the humps were no longer present, but instead, EDDs were more clearly visible in subendothelial and mesangial areas (Fig. 2F). Immunohistological staining of both NAPlr and plasmin receptors was negative (Fig. 2H and I).

## Diagnosis and clinical follow-up after the second biopsy

PSAGN in the resolving phase was the most likely diagnosis, but the possibility of C3GN was considered. After the patient was discharged, he kept canceling his regular visits. Two years after the first kidney biopsy, he progressed to end-stage renal disease (ESRD) and started hemodialysis.

After induction of dialysis, C3 nephritic factors (C3NeF)-IgG and anti-factor H (aFH) autoantibodies were measured in stored serum. Serum aFH autoantibodies and C3NeF-IgG were measured according to a previously published enzyme-linked immunosorbent assay method [6]. At the first biopsy, C3NeF were in the normal range (7.62%; normal, < 13.72%), but aFH antibodies were elevated (246.8 AU/mL; normal, < 100 AU/mL). The level of aFH antibodies was 200.6 AU/mL at the second biopsy (i.e., five months after the first biopsy) and 201.2 AU/mL 18 months after the first biopsy.

The final diagnosis was considered to be aFH antibody-positive C3GN secondary to PSAGN.

## Discussion and conclusions

PSAGN is an endocapillary proliferative acute glomerulonephritis that develops 1 to 3 weeks after an upper respiratory tract or skin infection. Kidney biopsy is characterized by diffuse endocapillary hypercellularity on LM and fine granular deposition of C3 on mesangial cells and the glomerular capillary tuft on IF. EM shows subepithelial humps on the GBM. Oda et al. identified NAPlr as a nephritogenic antigen released from streptococcal species that shows positivity in the region corresponding to where neutrophils have infiltrated the GBM in PSAGN patients. [4, 5]**.**

PSAGN used to be more common in children; however, in recent years its incidence has been increasing in adults with type 2 diabetes. The increased incidence in adults with type 2 diabetes is thought to be due to the increased risk of infection associated with skin disorders related to diabetic neuropathy and peripheral vasculopathy [1]. There is a report that ficolin-3-mediated complement lectin pathway activation and alternative pathway amplification were decreased in type 2 diabetic patients, suggesting diminished complement-mediated protection against bacterial infections in type 2 diabetic patients [7]. Adult-onset glomerulonephritis secondary to infections is now reported collectively as infection-related glomerulonephritis or postinfectious glomerulonephritis (PIGN), which includes PSAGN [1, 2]**.** As Nasr et al. reported, the renal prognosis of PIGN has been reported to be poor**.**[2] In our case, we assumed that type 2 diabetes influenced the development of PIGN and the poor renal prognosis.

The repeat kidney biopsies in our patient showed findings consistent with C3GN followed by PSAGN. This unique pathological course is another noteworthy feature of this case. Smith et al. reported that co-deposition of IgG and C3 is commonly observed in patients with PIGN and that C3-dominant glomerular deposition might represent a late stage of this disease [8]. The authors acknowledged the difficulty of distinguishing between PIGN and C3GN based on pathological features. The autoantibodies C3NeF are identified in 50% to 80% of patients with C3GN [8]**.** Sethi et al. evaluated 11 patients with features such as persistent proteinuria and hematuria or progression to ESRD, which were different from the usual features of PSAGN; they found that tests in seven patients were positive for C3NeF, but tests for aFH antibody were negative [9]. In our case, C3NeF was not found in the serum, but at both biopsies, the aFH antibody titers were high.

aFH antibody was initially found in patients with atypical hemolytic uremic syndrome (aHUS), but the relationship of this antibody with C3GN also has attracted attention [6, 10]. aFH antibodies prevent factor H from combining with C3. Consequently, circulating, unbound C3 could deposit in the glomerular capillary tufts, triggering glomerulonephritis by activating the alternative complement pathway. Dragon-Durey et al. reported that aFH antibodies were also detected in a small proportion of patients with C3GN [10]**.** When high levels of autoantibodies persist, functional deficiency of FH is induced, and predominant C3 deposits mediated by activation of the alternative complement pathway are exacerbated in renal histology. They emphasize that plasma exchange or immunosuppressive therapy is an effective treatment for patients with a high potency of this factor [10]. In our case, it is difficult to determine how long the aFH antibodies had been present, whether they were acquired after the infection that caused urinary tract infection, or whether aFH antibodies activated the alternative pathway despite the presence of type 2 diabetes mellitus, which decreased the pathway amplification [7]. Pilania RK et al. presented 2 cases of HUS. One was subjected to Shiga toxin-producing Escherichia coli HUS (STEC-HUS) with an unusual clinical course followed by thrombotic microangiopathy proven by kidney biopsy, and the other is atypical HUS caused by Plasmodium vivax. aFH antibodies were positive in both cases and they were both treated by plasmapheresis. [11] If aFH were positive congenitally or before infection, they would have developed HUS long before. Therefore, in our case, the infection triggered the activation of the alternative pathway, resulting in the generation of aFH antibodies and subsequent continuous activation of the pathway, leading from PSAGN to C3GN. Antibody-eliminating therapies may effectively suppress complement pathway activation and improve renal prognosis. However, this is only our speculation and the limitation of this case. Further studies are needed to determine when the antibodies are generated.

In conclusion, we experienced a case of C3 glomerulonephritis secondary to adult-onset PSAGN with diabetic nephropathy. This case indicates that type 2 diabetes may be a possible risk factor for PSAGN, and we speculate that the persistence of high titers of aFH antibodies may have caused C3GN secondary to PSAGN. Antibody-eliminating therapies such as plasma exchange therapy and immunosuppressive therapy may be worth trying in the future to improve renal prognosis in these patients. In this case, the other message was that when clinical features were not explained by the only first pathological diagnosis, such as diabetic nephropathy in this case, a repeat biopsy may reveal unexpected findings and should be considered.

## Data Availability

All data generated or analyzed during this study are included in this article, further inquiries can be directed to the corresponding author.

## Abbreviations

ASO: Anti-streptolysin O
C3GN: C3 glomerulonephritis
C3NeF: C3 nephritic factor
aFH antibody: Anti-factor H antibody
CRP: C-reactive protein
DM: Diabetic mellitus,
EDD: Electron-dense deposit,
LM: Light microscopy
IF: Immunofluorescence
EM: Electron microscopy
GBM: Glomerular basement membrane
NAPlr: Nephritis-associated plasmin receptor
PSAGN: Poststreptococcal acute glomerulonephritis,
IRGN: Infection-related glomerulonephritis,
PIGN: Postinfectious glomerulonephritis,
aHUS: Atypical hemolytic uremic syndrome
